# Dental Chemistry and Metallurgy
*Dental Chemistry and Metallurgy, 2nd edition, revised. By Clifford Mitchell, M.D. 410 pp. (W. P. Keener, Chicago.)

**Published:** 1891-02-21

**Authors:** 


### THE PRACTITIONER'S BOOKSHELF.

### DENTAL CHEMISTRY AND METALLURGY.*

This work, which has now reached its second edition, is intended as a practical working manual for the dental student. We consider it very conveniently arranged, and a valuable book for reference. Apart from the occasional discrepancy of the United States with the British Pharmacopœia, it is a valuable addition to our chemical literature, and a most useful book for a dental student.

* Dental Chemistry and Metallurgy, 2nd edition, revised. By Clifford Mitchell, M.D. 410 pp. (W. P. Keener, Chicago.)

